# Mfn2 ablation causes an oxidative stress response and eventual neuronal death in the hippocampus and cortex

**DOI:** 10.1186/s13024-018-0238-8

**Published:** 2018-02-01

**Authors:** Sirui Jiang, Priya Nandy, Wenzhang Wang, Xiaopin Ma, Jeffrey Hsia, Chunyu Wang, Zhenlian Wang, Mengyue Niu, Sandra L. Siedlak, Sandy Torres, Hisashi Fujioka, Ying Xu, Hyoung-gon Lee, George Perry, Jun Liu, Xiongwei Zhu

**Affiliations:** 1 Department of Pathology, Case Western Reserve University, 2103 Cornell Road, Cleveland, OH USA; 2 Department of Neurology, the second Xiangya Hospital, Central South University, Changsha, Hunan People’s Republic of China; 3 School of Pharmaceutical Engineering & Life Sciences, Changzhou University, Changzhou, Jiangsu 213164 China; 4 Department of Neurology & Institute of Neurology, Ruijin Hospital, Shanghai Jiao Tong University School of Medicine, Shanghai, China; 5 Electron Microscopy Core Facility, Case Western Reserve University, Cleveland, OH 44106 USA; 6 Department of Pharmaceutical Sciences, School of Pharmacy and Pharmaceutical Sciences, State University of New York at Buffalo, Buffalo, NY 14222 USA; 7 Department of Biology, The University of Texas at San Antonio, One UTSA Circle, San Antonio, TX 78249 USA

## Abstract

**Background:**

Mitochondria are the organelles responsible for energy metabolism and have a direct impact on neuronal function and survival. Mitochondrial abnormalities have been well characterized in Alzheimer Disease (AD). It is believed that mitochondrial fragmentation, due to impaired fission and fusion balance, likely causes mitochondrial dysfunction that underlies many aspects of neurodegenerative changes in AD. Mitochondrial fission and fusion proteins play a major role in maintaining the health and function of these important organelles. Mitofusion 2 (Mfn2) is one such protein that regulates mitochondrial fusion in which mutations lead to the neurological disease.

**Methods:**

To examine whether and how impaired mitochondrial fission/fusion balance causes neurodegeneration in AD, we developed a transgenic mouse model using the CAMKII promoter to knockout neuronal Mfn2 in the hippocampus and cortex, areas significantly affected in AD.

**Results:**

Electron micrographs of neurons from these mice show swollen mitochondria with cristae damage and mitochondria membrane abnormalities. Over time the Mfn2 cKO model demonstrates a progression of neurodegeneration via mitochondrial morphological changes, oxidative stress response, inflammatory changes, and loss of MAP2 in dendrites, leading to severe and selective neuronal death. In this model, hippocampal CA1 neurons were affected earlier and resulted in nearly total loss, while in the cortex, progressive neuronal death was associated with decreased cortical size.

**Conclusions:**

Overall, our findings indicate that impaired mitochondrial fission and fusion balance can cause many of the neurodegenerative changes and eventual neuron loss that characterize AD in the hippocampus and cortex which makes it a potential target for treatment strategies for AD.

**Electronic supplementary material:**

The online version of this article (10.1186/s13024-018-0238-8) contains supplementary material, which is available to authorized users.

## Background

Alzheimer Disease (AD) is a multifactorial, age-related neurodegenerative disease of the elderly that leads to progressive memory loss, impairments in behavior, language, visual-spatial skills and ultimately death. The disease is characterized by a progressive neuronal loss and accumulation of extracellular senile plaques composed of amyloid beta (Aß) and intracellular neurofibrillary tangles (NFT) composed of hyperphosphorylated tau in selective brain areas such as hippocampus and cortex [1]. According to 2015 World Alzheimer’s Report, 47.5 million people had AD-related dementia worldwide, including 5.4 million Americans, and projected the numbers to rise to 75.6 million by 2030 and to 131.5 million by 2050. Over 9.9 million new cases of AD-related dementia are diagnosed every year worldwide [2]. Dementia has a huge economic impact on our society and the estimated total healthcare cost of dementia worldwide in 2015 was $818 billion. Currently, there are no drugs or agents available to treat or to prevent AD.

Several decades of intensive research have revealed that multiple cellular changes have been implicated during the course of AD including widespread oxidative damage, extensive neuroinflammation, and aberrant cytoskeletal alteration among which mitochondrial dysfunction is an early prominent feature in susceptible neurons in the brain from AD patients and models of AD, and likely plays a critical role in the pathogenesis of AD [1, 3, 4]. Indeed, a reduced rate of brain metabolism preceding functional impairment is one of the best documented abnormalities in AD which is likely due to deficiency in several key enzymes of oxidative metabolism including cytochrome oxidase (COX) that have been consistently demonstrated in AD [3, 4]. However, mechanisms underlying mitochondrial dysfunction in AD remains elusive.

Mitochondria are dynamic organelles that continuously fuse with each other to form larger tubular networks and divide into smaller structures, a process regulated by the balance of fission/fusion machinery mainly involving several large GTPases: mitochondrial fission is regulated by cytosolic protein DLP1 which translocates to mitochondria during fission while mitochondrial fusion is regulated by mitofusin 1 and 2 on the outer mitochondrial membrane and OPA1 on the inner mitochondrial membrane [5]. The delicate balance between mitochondrial fission and fusion is crucial in the maintenance of healthy population of mitochondria and proper mitochondrial distribution, morphology and function, disruption of which causes human diseases, especially neurological diseases [5]. For example, thus far, genetic mutations were found in Mfn2 and reported to be associated with Charcot-Marie-Tooth (CMT) disease, the most common inherited neurological disorders, accounting for up to 20 to 30% of all axonal CMT type 2 cases, to a lesser degree, also be associated with optic atrophy, clinical signs of first motor neuron involvement, and early onset stroke [6–8]. Increasing evidence suggested that an abnormal mitochondrial dynamics is likely involved in the mitochondrial structural damage and dysfunction in AD: several groups demonstrated that overexpression of familial AD-causing amyloid precursor protein (APP) mutants or exposure to soluble oligomeric Aβ caused changes in the expression of mitochondrial fission and fusion proteins and profound mitochondrial fragmentation in neuronal cells which led to ultrastructural damage to mitochondria and mitochondrial dysfunction along with neuronal deficits such as synaptic abnormalities in vitro [9–14]. Aβ-induced changes in mitochondrial dynamics and distribution are early events in vivo in *Drosophila* models [15, 16]. Abnormal mitochondrial distribution and round, swollen, and damaged mitochondria consistent with enhanced fragmentation are also documented in AD mouse models [17–19]. Fibroblasts from AD patients and AD cybrid cell models also demonstrated abnormal mitochondrial dynamics and dysfunction [20, 21]. Indeed, ultrastructural deficits and abnormal distribution of mitochondria were evident in pyramidal neurons in AD brain [9]. Although no specific mutations in fusion genes were associated with AD, studies from several groups consistently demonstrated a significantly reduced expression of large GTPases involved in fusion (i.e., Mfn1, Mfn2 and OPA1) in the brain of AD patients, implicating that mitochondrial fragmentation, largely due to disrupted mitochondrial fusion, likely occurs in the susceptible neurons in AD brain [12, 22, 23]. However, whether disruption of mitochondrial fusion causes mitochondrial deficits and neurodegeneration in AD-afflicted brain areas has not been determined. To address the causal role of disrupted mitochondrial fusion in neurodegeneration and other AD-related deficits in AD-afflicted brain areas, we disrupted mitochondrial fusion by knocking out Mfn2 in the hippocampus and cortex and found that ablation of Mfn2 caused neuronal degeneration in vivo in a temporal order starting with significant mitochondrial fragmentation and dysfunction and increased oxidative stress, followed by cytoskeletal alterations and inflammatory response that culminates in eventual neuronal death, all features characterizing AD, thus establishing the critical role of mitochondrial fragmentation in mitochondrial dysfunction and neuronal degeneration and the pathogenesis of AD.

## Methods

### Transgenic mice

Experimental mice were generated by crossing B6.Cg-Tg(Camk2a-cre)T29-1Stl/J mice (Jackson Laboratory) with B6.129(Cg)-*Mfn2*^*tm3Dcc*^/J mice (Mfn2^loxP^). PCR of genomic DNA was used for routine genotyping of offspring to detect the presence of the CAMKCre gene and the Mfn2^loxP^ gene. We chose these mice since the Cre-recombinase was shown to be expressed in the forebrain and hippocampus, highest in the CA1 layer. Mice were housed under standard conditions and all animal studies were performed following an approved protocol through the Case Western Reserve University IACUC board. At various ages, mice were perfused with saline and brain tissue collected and bisected with one half frozen (with brain areas cortex, hippocampus, cerebellum and brainstem area separated) and the other half fixed in 10% buffered formalin. For this constitutive expression study, Mfn2 cKO mice from ages 4, 8, 12, 18, 28, and 78 weeks (at least *n* = 3 per age group) and age-matched control mice (no Cre+, at least *n* = 3 per age group) were collected.

### PCR

To confirm recombination of the floxed gene, DNA was extracted from frozen samples of cortex, hippocampus, cerebellum and brainstem using the Wizard genomic DNA kit. PCR was performed using primers designed to amplify a 240 bp band only after Cre recombination has occurred.

### Immunoblotting

For Western blot analysis frozen dissected hippocampal and cortical tissues were individually homogenized in 10X volume Cell lysis buffer (Cell signaling) with added protease and phosphatase inhibitors (Roche) and centrifuged at 14,000 g for 20 min in a refrigerated microcentrifuge. Protein concentration of the supernatant was determined using BCA protein assay. Proteins, 10 μg per lane (specified in figure legends), were resolved with SDS-PAGE and transferred to Immobilon (Millipore). Blots were blocked in 10% nonfat milk for 1 h and probed with primary antibodies overnight. After washing 5X in TBS-tween, HRP-conjugated secondary antibodies (Cell Signaling) were applied for 1 h, rinsed again and bands detected using ECL (Santa Cruz or Millipore). Loading control was anti-actin (Millipore). Bands were quantified using QuantityOne (Biorad) or ImageJ.

### Immunohistochemistry

Formalin fixed brain samples were embedded in paraffin and 6 μm sections were cut using a microtome and placed on coated slides. Immunohistochemistry was performed using the peroxidase anti peroxidase method as previously described [24]. Briefly, sections were deparaffinized in 2 changes of xylene, dehydrated through descending series of ethanol, and finally into Tris buffered saline (TBS: 50 mM Tris, 150 mM NaCl, pH = 7.6). Endogenous peroxidase was removed with a 30-min incubation in 3% H_2_O_2_. Antigen retrieval using citrate buffer and pressure cooking (Biocare) was performed for most IHC experiments. After blocking for 30 min in 10% normal goat serum (NGS), primary antibodies were applied and incubated overnight at 4 °C. Species specific secondary antibodies and PAP complexes were applied and after 3 changes of tris buffer, the sections were developed with DAB (Dako) and slides were rinsed in dH2O, dehydrated and coverslipped with Permount. Antibodies used were directed against Cre-recombinase (Millipore), GFAP (Invitrogen), MAP2 (Millipore), NeuN (Millipore), COXI (Molecular Probes), HO-1 (Enzo), iba1 (Wako), AT8 (Thermo Fisher), and mitochondria complex cocktail (Abcam).

To detect protein carbonyls, the dinitrophenol binding -assay was also performed. After tissue sections were rehydrated, 50 ul of DNP solution was applied (20 mM DNP, 0.5% TFA, 92.5% DMSO) and incubated for 15 min at 37 °C. The sections were rinsed with changes of acetic acid until no yellow color could be further removed. After rinsing thoroughly with water and then with TBS, sections were blocked and DNP detected with rabbit antibody to DNP (1/10,000). For western blot analysis, samples were first denatured and then treated with DNP solution following manufacturer’s instructions (Millipore), sample buffer directly added and the entire sample run on the gel.

TUNEL method was used to label cells undergoing apoptosis following manufacturer’s instructions (Roche). After TUNEL method, the sections were stained with DapI and mounted with Fluoromount (SouthernBiotech) and FITC-labelled apoptotic cells were imaged on a Zeiss Axiophot.

### Image quantification

Images were acquired on a Axiophot with a axiocam (Zeiss). Quantification was performed on these images using Axiovision software and either the number of cells stained per area, or density of stained structures was determined using Axiocam image analysis software. To measure hippocampal size, H&E stained sections from brain areas collected from Bregma 1.08–1.8, representing the complete hippocampus and similar ventricle presentation were imaged and measured in a blinded fashion. To measure total cortical size, sections from similar levels were imaged at the same magnification, the cortical area cropped using Photoshop and the area was quantified.

### Electron microscopy

For electron microscopic analysis, brain samples from control and KO mice were collected and fixed as previously described following brain dissection [19]. Brain slices of about 1 mm thick were made and small areas of the CA1 region of the hippocampus and cortex were sampled and embedded in Epon. Semithin sections were prepared and stained with toluidine blue to clearly note the CA1 regions. Images of pyramidal neurons from the CA1 region or cortex with initial segment of axon visible were obtained. Mouse genotype was blinded to the electron microscopist. Mitochondria parameters were quantified using Image J and included aspect ratio (length/width) and size (area in μm^2^).

### Mitochondrial oxygen consumption measurement

The real-time measurement of oxygen consumption rate (OCR) in synaptic mitochondria in synaptosomes was performed using the Seahorse XF24 Analyzer (Seahorse Bioscience, North Billerica, MA), according to the manufacturer’s instructions. If needed, ATP synthase inhibitor oligomycin (1 μM), uncoupler FCCP (4 μM) and complex I inhibitors antimycin A (1 μM) and rotenone (1 μM) were injected sequentially. After measurement, cells and synaptosomes were lysed and OCR data was normalized by total protein as previously described [24].

## Results

### Conditional knockout of Mfn2 in the forebrain

**Fig. 1:**
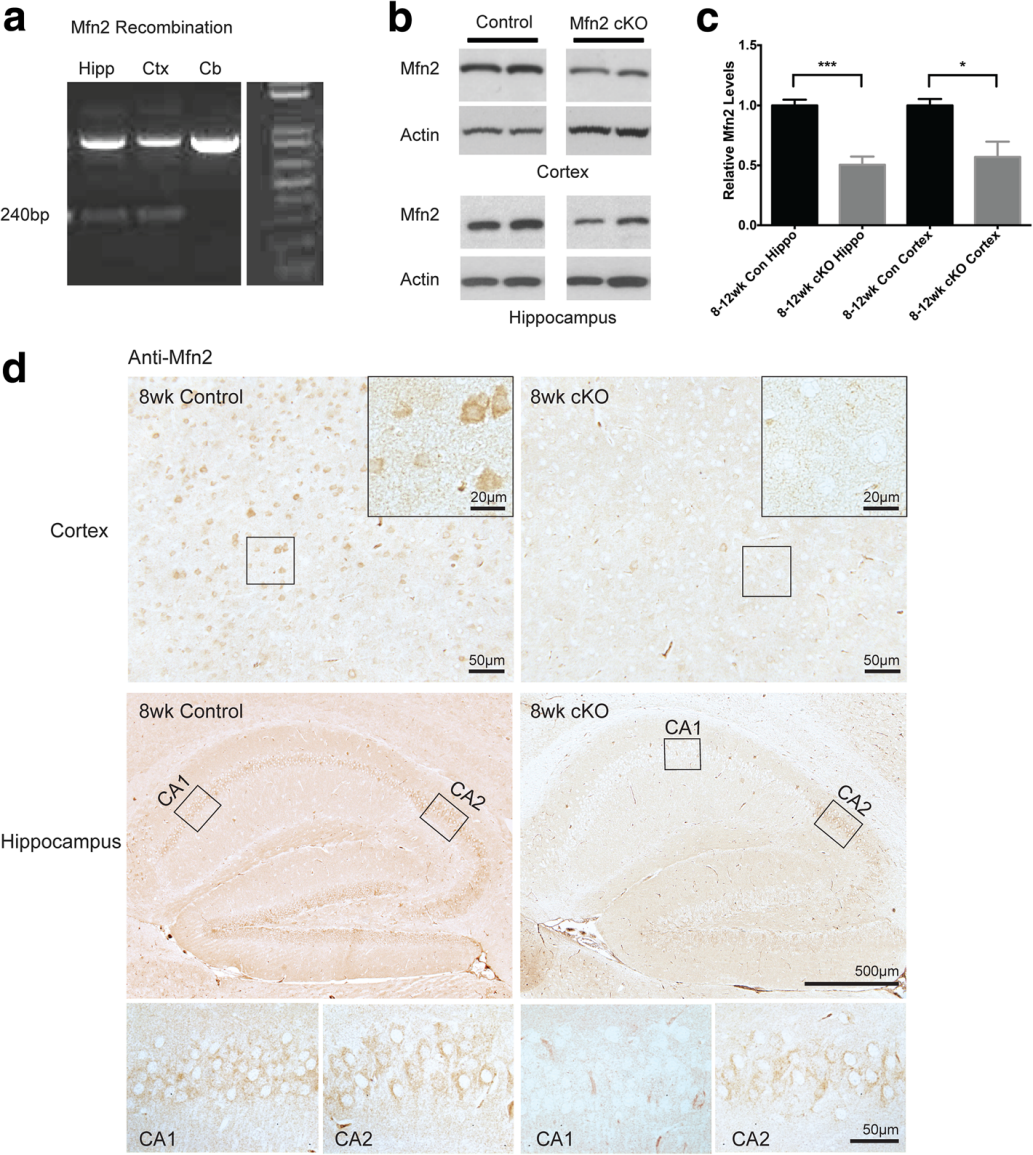
Cre-mediated ablation of Mfn2 expression in the hippocampus and cortex of Mfn2 cKO mice. **a** Cre-mediated recombination and excision of floxed Mfn2was analyzed by genomic DNA PCR. Cre-mediated ablation of Mfn2 was found in the hippocampus (Hip) and cerebral cortex (Cx) but not in cerebellum (Cb). Western blot (**b**, representative from animals of 8–12 weeks of age) and quantification analysis (**c**) of brain homogenates found the Mfn2 protein levels were reduced in Mfn2 cKO mice (*N* = 7) compared to control mice (*N* = 6) in both the hippocampus and the cortex. Actin was used as an internal loading control. Data are means ± SEM, student’s t-test, *P < 0.05, ***P < 0.001. **d** Mfn2 immunostaining of brain sections from 8-week-old control and Mfn2 cKO mice showed a loss of Mfn2 protein in both cortical (boxed area was enlarged in the upper-right corner) and CA1 hippocampal neurons but not CA2 hippocampal neurons (boxed areas were enlarged below)

Mfn2 levels are significantly reduced in AD and likely contribute to increased mitochondrial fragmentation [12, 23]. To assess the causal relationship between disrupted mitochondrial fusion and AD-related deficits in the brain areas affected by AD, we utilized a genetic approach by crossing the Mfn2 conditional knockout mice (Mfn2^loxP/loxP^) with CaMKII-Cre mice, in which the Cre recombinase is expressed in the forebrain and result in specific ablation of Mfn2 in selective neurons in the hippocampus and cortex, brain areas that are heavily afflicted in AD. Genomic DNA PCR analysis confirmed Cre-mediated recombination and excision of floxed Mfn2 (Fig. 1a) in the hippocampus and cortex but not in the cerebellum in the homozygous knockout mice (CaMKII-Cre^+/−^/Mfn2^loxP/loxP^, hereafter referred to as Mfn2 cKO). Western blot revealed that Mfn2 protein levels were significantly reduced in both the hippocampus and cortex of the homozygous Mfn2 cKO mice, compared to control mice (Fig. 1b), at age of 8 to 12 weeks (Fig. 1c). To confirm the specific loss of Mfn2 protein in the selective neurons of the hippocampus and cortex, we performed immunocytochemical study in the 8 week old mice brains (Fig. 1d, Additional file 1: Figure. S1). Consistent with previous studies using CaMKII-Cre mice to ablate the floxed target gene [25], there was a selective loss of the Mfn2 protein (Fig. 1d) in the cortical neurons and in pyramidal neurons in the CA1 region but not CA2 region of the hippocampus. We also determined the expression of other fission and fusion proteins in the Mfn2 cKO mice by western blot and found no significant changes in the expression of DLP1 or OPA1 in the Mfn2 cKO mice at 8 weeks of age (Additional file 1: Figure. S1). The Mfn2 cKO mice develop and grow normally and display normal fertility with no gender differences.

### Mfn2 knockout caused mitochondrial ultrastructural deficits and abnormal distribution

**Fig. 2:**
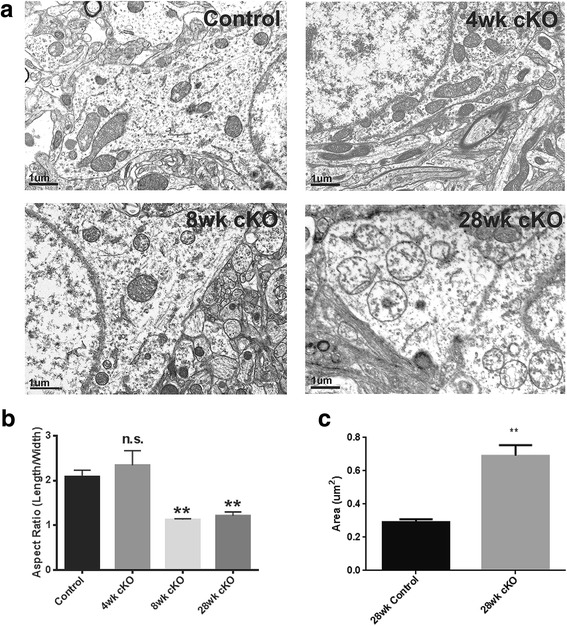
Mfn2 ablation caused mitochondrial fragmentation and ultrastructural damage in the hippocampus in vivo as evidenced by electron microscopic analysis. **a** Representative electron micrographs of CA1 neuron from hippocampus of control (28-week-old) (upper left) and Mfn2 cKO at different ages as indicated. **b** Quantification of aspect ratio demonstrated significant mitochondrial fragmentation in 8 and 28 week old Mfn2 CKO mice. **c** Quantification of mean area of mitochondria demonstrated significantly enlarged mitochondrial size in 28 week old Mfn2 cKO mice compared to control mice. Data are means ± SEM Student’s t-test, *P < 0.05

To visualize the impact of disruption of mitochondrial fusion by Mfn2 ablation on mitochondrial morphology and distribution in the hippocampus, electron microscopy was performed to examine mitochondria in the CA1 pyramidal neurons. Tubular mitochondria with the regular, accordion-like folds of cristae without any notable abnormalities were found in the CA1 pyramidal neurons in both the youngest (i.e., 4 weeks) (not shown) and the oldest littermate control mice examined (i.e., 28 weeks) (Fig. 2a). Similarly, at the age of 4 weeks, no obvious abnormalities in the appearance of mitochondria were noted in the Mfn2 cKO mice (Fig. 2a). However, dramatic changes in mitochondrial morphology and cristae organization were noted in the CA1 pyramidal neurons of Mfn2 cKO mice by the age of 8 weeks (Fig. 2a): mitochondria appeared rounder and swollen with broken cristae, many of them also exhibiting multilamellar appearance and vacuolation. It became more severe in the neurons of 28 week old Mfn2 cKO mice where extreme loss of internal cristae structure were frequently seen (Fig. 2a). Quantification of all mitochondria in 4 to 5 neurons per mouse find that the average mitochondrial aspect ratio (length/width of each mitochondria) was unchanged in 4 week Mfn2 cKO mice but became significantly decreased in 8 week and 28 week Mfn2 cKO mouse to a number that close to 1, consistent with the rounder shape in appearance (Fig. 2b). Importantly, in 28 week Mfn2 cKO mice, mitochondrial size was significantly increased and the mean size almost doubled compared to the control mice, confirming the significant swollen phenotype (Fig. 2c). Notably, these mitochondria almost completely lost their cristae structures.

**Fig. 3:**
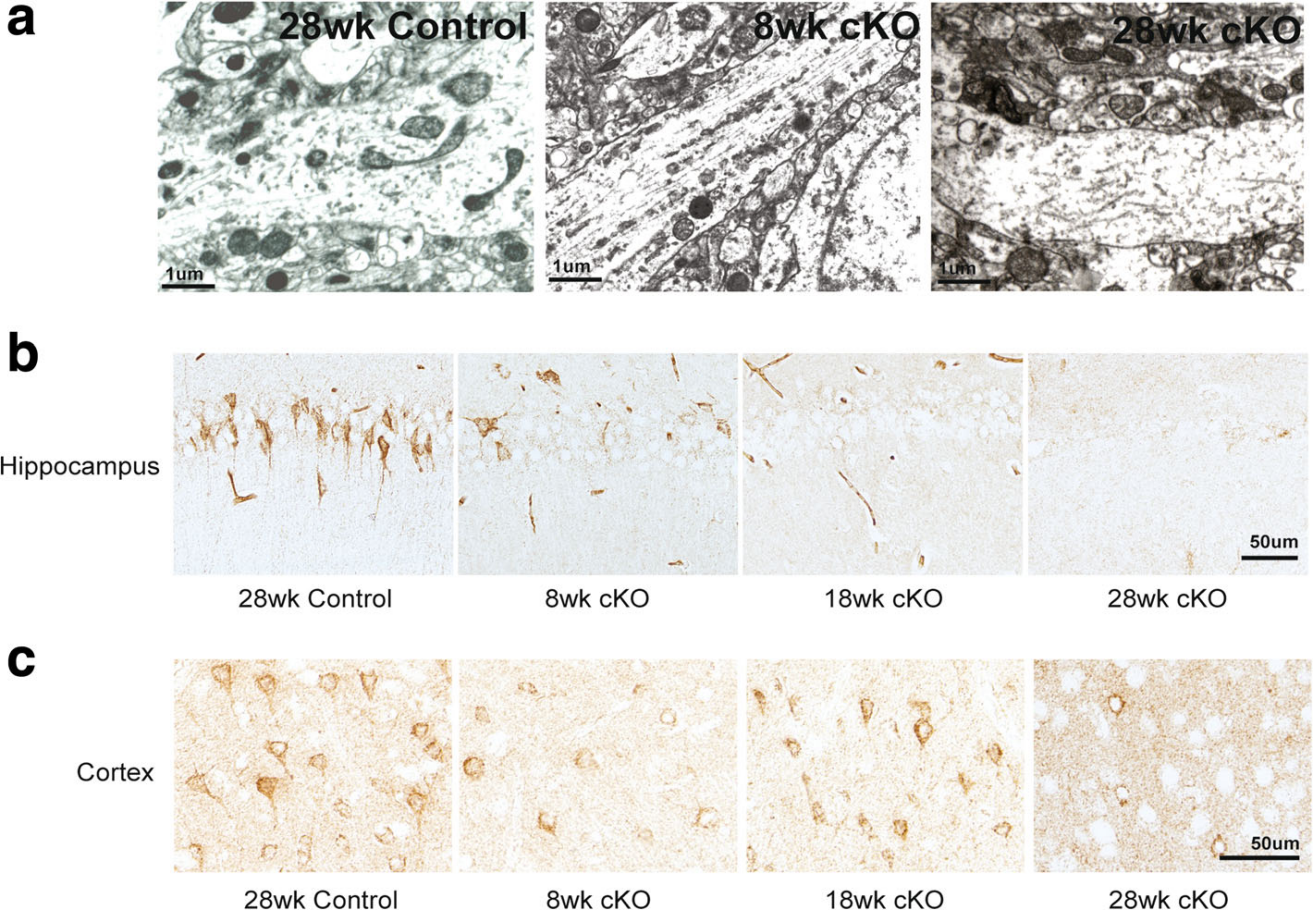
Mfn2 ablation caused abnormal mitochondrial distribution in vivo. **a** Rerpresentative electron micrographs of a segment of axon of CA1 neuron from hippocampus of control and Mfn2 cKO mice. Immunohistochemistry using OXPHOS cocktail antibody to stain mitochondria finds many neurons in the hippocampus (**b**) and frontal cortex (**c**) exhibiting staining throughout the cell body and processes in the control mice. However, at as early as 8 weeks, a striking loss of neuronal process immunolocalization in the CA1 and cortical neurons occurs and becomes more apparent with aging

Additionally, mitochondria in neurons from control mice are generally distributed evenly along the entire neuronal processes, however, mitochondria in the processes of 8 week Mfn2 cKO mice were far less numerous, demonstrating abnormal mitochondrial distribution. This abnormal distribution of mitochondria is further exacerbated in the 28 week Mfn2 cKO mice (Fig. 3a). Consistently, immunohistochemistry using antibodies against mitochondria complex proteins demonstrate specific changes in the staining pattern that also confirmed abnormal mitochondrial distribution in Mfn2 cKO mice: mitochondria are distributed throughout the neuronal cell body and along the processes in control mice at all ages examined, but instead accumulate mainly in cell body leaving processes largely devoid of mitochondria staining in both the hippocampus CA1 neurons and cortical neurons in as early as the 8 week old Mfn2 cKO mice (Fig 3b).

### Mfn2 knockout caused mitochondrial dysfunction

**Fig. 4:**
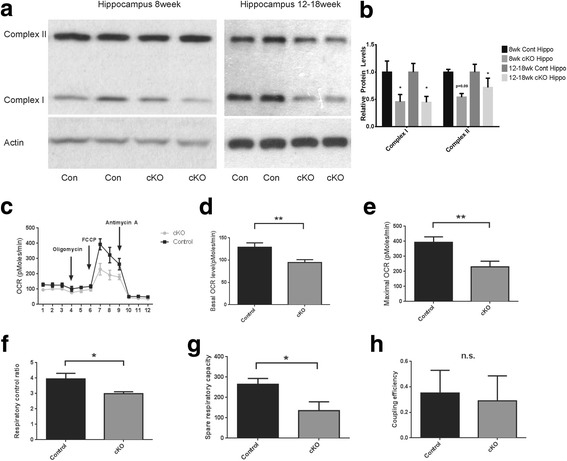
Mfn2 ablation caused mitochondrial dysfunction in the brain of Mfn2 cKO mice. Representative western blots (**a**) and quantification analysis (**b**) showed changes in the protein levels of key subunits of respiratory complex I (anti-NDUFB8), and II (anti-SDHB) in the hippocampal and cortical tissues of Mfn2 cKO mice since 8 week of age. (**c**) Respiratory activity of synaptic mitochondria freshly isolated from hippocampus of 8-week-old Mfn2 cKO mice and control mice was analyzed by Seahorse XF Assay. Oxygen consumption (OCR) rate was measured before and after sequential exposure to oligomycin (inhibits ATP synthase, blocks oxygen consumption related to ATP synthesis), FCCP (uncoupler to assess maximal OCR), and antimycin A/rotenone (blocks electron flux through both complex I and II). Quantification of basal (**d**) and maximal (**e**) OCR of control and Mfn2 cKO mice. Respiration control ratio (F) (OCR_FCCP_/OCR_Oligomycin_), Spare respiratory capacity (**g**) (OCR_FCCP_-OCR_Basal_) and Coupling efficiency (H) ([OCR_Basal_ -OCR_Oligomycin_]/ OCR_Basal_) were calculated after subtracting the non-mitochondrial respiration (OCR_Antimycin_ _A/rotenone_). Data are means ± SD of 3 mice. Statistics: Student’s t test. *p < 0.05 and **p < 0.01 compared with control

We then characterized whether and how mitochondrial function is affected in Mfn2 cKO mice. To examine changes in the expression of proteins involved in electron transport chains, we performed western blot analysis of the hippocampal tissues from the control and Mfn2 cKO mice. Starting at 8 weeks old and continuing with age, a significant loss of complex I is seen in the hippocampus of the Mfn2 cKO mice (Fig. 4a, b). There is a decrease trend in Complex II which becomes significant at 12 to 18 weeks of age. No changes were found in either Complex III or V even by 28 weeks (data not shown). To determine the impact of Mfn2 cKO on mitochondrial function, mitochondrial respiration was determined in freshly isolated synaptic mitochondria from hippocampus of 8 weeks old Mfn2 cKO mice and their littermate controls using a Seahorse XF24 extracellular flux analyzer. Both basal and maximal oxygen consumption rate (OCR) along with spare respiratory capacity (difference between basal and maximal respiratory capacity, critical for neuronal bioenergetics under stress) were significantly reduced in the synaptic mitochondrial from Mfn2 cKO mice as compared with littermate control mice (Fig. 4c-e, g). Respiration control ratio calculated from OCR measurement after FCCP and oligomycin treatments was also significantly decreased in Mfn2 cKO mitochondria but mitochondrial coupling efficiency remains unchanged (Fig. 4f, h).

### Mfn2 cKO caused severe neurodegeneration in the hippocampus and cortex

**Fig. 5:**
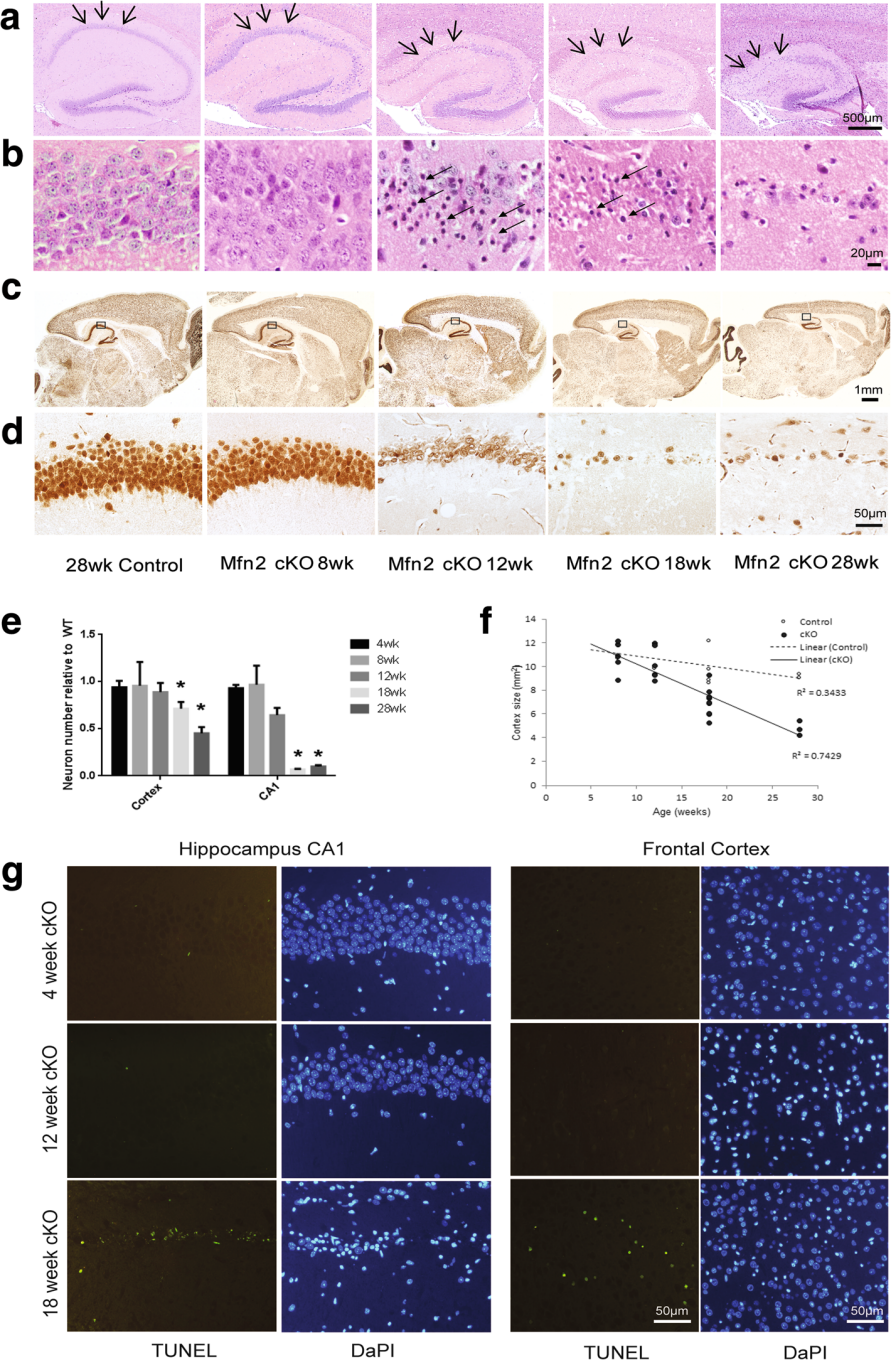
Mfn2 ablation caused neurodegeneration in the hippocampus and cortex in vivo. **a** Representative H&E stains of the hippocampus revealed an obvious pattern of hippocampus neuronal degeneration in the CA1 area of Mfn2 cKO mice with aging, yet the dentate gyrus and CA2 remained intact across all ages. **b** Enlarged picture of H&E staining of the CA1 neurons revealed a shrunken appearance of nuclei of many CA1 neurons in the Mfn2 cKO mice starting at 12 weeks of age. **c** Using low magnification images immunostained using NeuN, the cortical area also becomes shrunken with age (**c**) and quantification reveals this is a significant correlation (F, *p* < 0.001). **d** Enlarged picture of the boxed areas in (**c**) of the NeuN staining of the CA1 neurons. Quantification revealed a significant loss of neurons in both the cortex and CA1 of the hippocampus at 18 weeks (**e**). Neuronal apoptosis detected by TUNEL assay was only seen at 18 weeks in both the hippocampus and cortex (**g**). No apoptotic cells were present in any of the 4 or 12 week old cKO mice, but the number of CA1 neurons is reduced in the 12 week old mice compared to the 4 week old mice seen in the DapI images, reflecting the cellular changes noted by H&E staining at 12 weeks of age

H&E stains revealed apparent hippocampal neuronal degeneration in the CA1 area with aging in Mfn2 cKO mice compared with age-matched littermate control mice (Fig. 5a, b): Neurons in the hippocampus in Mfn2 cKO mice up to 8 weeks appeared normal; starting at 12 weeks of age, many of the CA1 neuronal nuclei exhibited a shrunken appearance although no significant changes in the number of neurons were noted in Mfn2 cKO mice. Mfn2 cKO mice at ages 18 weeks demonstrated almost complete loss of hippocampal neurons, yet the dentate gyrus and CA2 neurons remained intact across all ages. The specific degeneration of neurons in the Mfn2 cKO mice was demonstrated by NeuN staining (Fig. 5c, d). Neuronal number in the hippocampus CA1 and CA2 regions quantified using the sections stained for NeuN revealed that the number of pyramidal neurons in the CA2 region remained constant with age (not shown), yet the number of pyramidal neurons in the CA1 region showed a trend towards decrease at 12 weeks of age which became significant at age 18 weeks (Fig. 5e). It appears that the majority cell death occur at the age 18 weeks and not much further cell death occur in 28 weeks (Fig. 5e). Indeed, the hippocampus size was also reduced significantly with age in the Mfn2 cKO mice but not in littermate control mice (data not shown).

Examination of the entire cortical region of sections stained with NeuN reveals severe shrinkage of the cortex in the Mfn2 cKO mice (Fig. 5c). In fact, quantification of the entire cortical area shows a slight but significant decrease in the cortical size in control mice with aging which became much more severe and significant in the Mfn2 cKO mice (Fig. 5f). Higher magnification images of the cortical region proximal to the hippocampus using Nissl staining showed the neuronal layers became disorganized with many nuclei disfigured and less uniform at 28 weeks of age (Additional file 1: Figure S2). The total number of NeuN-positive neurons in the cortex decreased significantly since 18 weeks of age and further decreased at 28 weeks of age (Fig. 5e).

TUNEL staining revealed apoptosis only at 18 weeks of age (Fig. 5g) in both the CA1 region and in the cortex of the Mfn2 cKO mice. No apoptotic cells were evident in any of the 12 week old Mfn2 cKO mice. DapI staining reveals some neuronal loss in the CA1 in the 12 week mice compared to the control mice (not shown) or 4 week old Mfn2 cKO mice (Fig. 5g), which correlates with the appearance of shrunken nuclei seen with H&E staining shown in Fig. 5b at this age.

### Mfn2 knockout caused increased oxidative stress

**Fig. 6:**
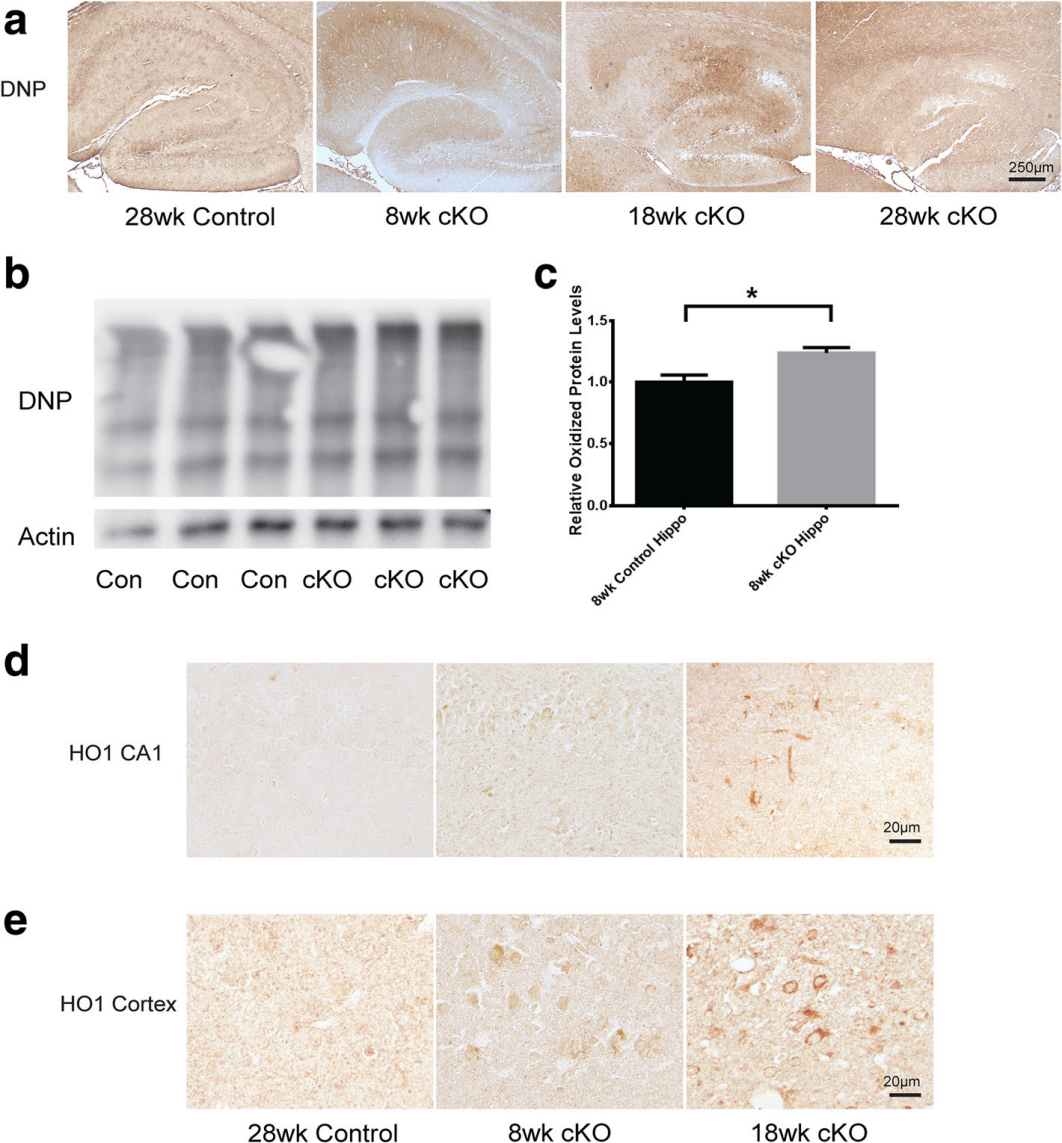
Mfn2 ablation caused increased oxidative stress in the hippocampus and cortex in vivo. **a** Representative of DNP adduction immunochemical assay to detect protein oxidation in the hippocampus of Mfn2 cKO mice and control mice. Representative western blots (**b**) and quantification analysis (**c**) showed significant increase in protein oxidation in 8-week-old Mfn2 cKO mice compared to age-matched control mice using the DNP assay. Actin was used as an internal loading control. (Data represent mean ± SEM, Student’s *t*-test, *P < 0.05). **d**-**e** Representative immunohistochemistry of anti-HO-1, an inducible antioxidant enzyme, in the hippocampus (**d**) and cortex (**e**) of Mfn2 cKO mice and control mice

Widespread oxidative stress is an early and prominent feature of AD which is believed plays a role during neurodegeneration [26]. Immunocytochemical studies revealed increased protein oxidation as demonstrated by enhanced DNP immunostaining suggestive of increased protein carbonyls as early as 8 weeks in the Mfn2 cKO mice which became more prominent with age (Fig. 6A). Indeed, by a DNP Oxyblot, protein carbonyls were significantly increased at 8 weeks in the hippocampus (Fig. 6b, c). These findings are corroborated through immunostaining of brain tissue for another oxidative marker, the antioxidant enzyme hemoxygenase-1 (HO-1): The pyramidal neurons demonstrate progressively increased levels of HO-1 in the Mfn2 cKO hippocampus and cortex since the age of 8 weeks (Fig. 6d, e).

### Mfn2 knockout caused increased neuroinflammation

**Fig. 7:**
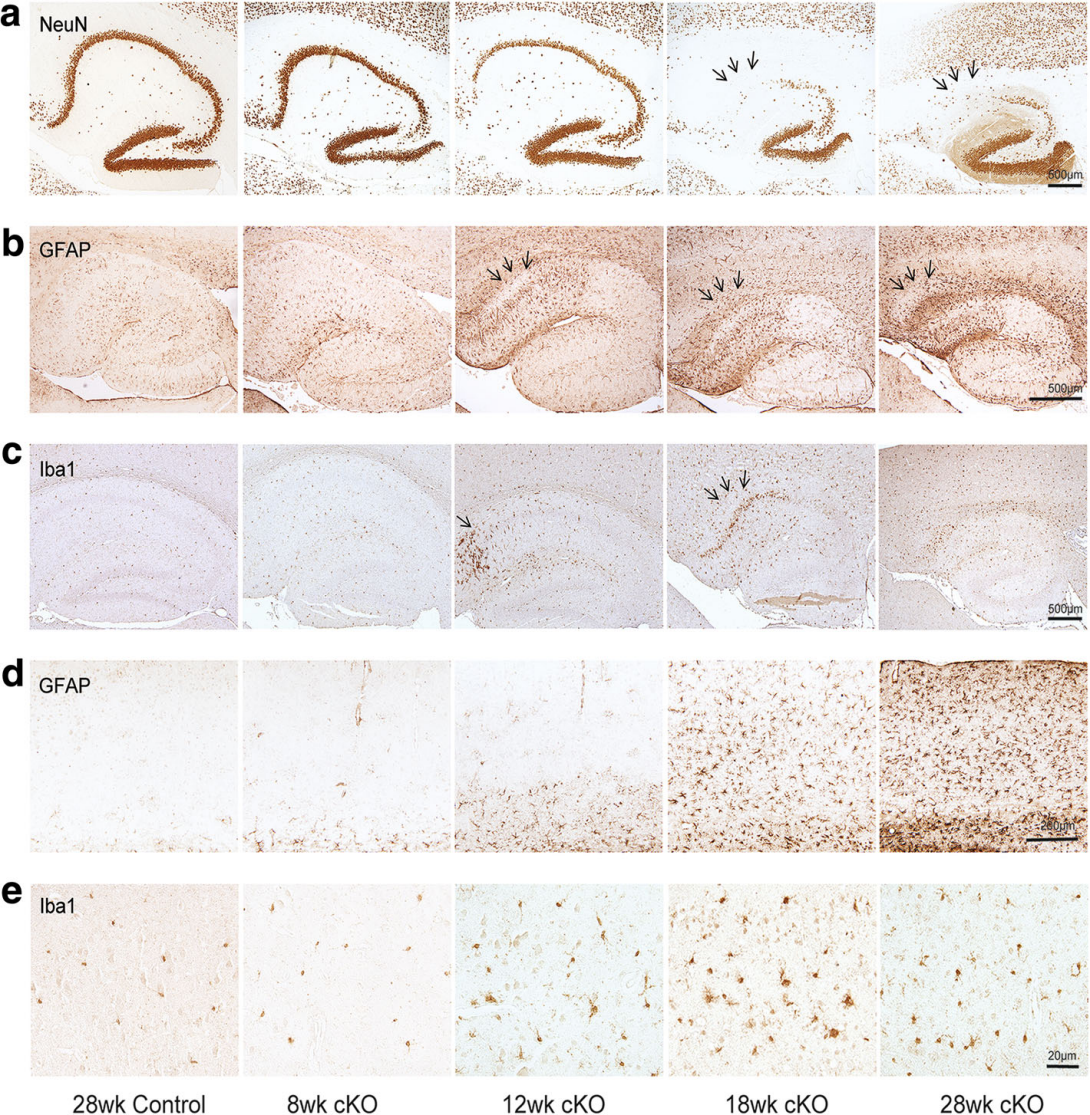
Mfn2 ablation caused increased neuroinflammation in hippocampus and cortex in vivo. **a** Representative immunohistochemistry of anti-NeuN antibody in the hippocampus of Mfn2 cKO and control mice for immediate comparison with the neuroinflammation marker. **b**-**e** Representative immunocytochemistry of anti-GFAP antibody in the hippocampus (**b**) and cortex (**d**) and anti-Iba1 antibody in the hippocampus (**c**) and cortex (**e**) of the Mfn2 cKO mice and control mice

Increased inflammation is another characteristic of AD [27]. Increased gliosis was found in the specific brain regions that displayed the neuronal degeneration and loss in older Mfn2 cKO mice (Fig. 7). Some increased GFAP-immunostaining began to appear in the CA1 region of hippocampus of Mfn2 cKO mice starting at 8 weeks of age which became prominent and significantly increased since 12 week of age (Fig. 7B and Additional file 1: Figure S3). The CA2 region remained spared from astrocyte accumulation in the Mfn2 cKO mice even at age 28 weeks (Fig. 7b). In the cortex, GFAP-positive astrocytes accumulate progressively with age appearing first in neuronal layer V/VI at 12 weeks and then encompassing all cortical neuronal layers by 18 weeks of age (Fig. 7d).

Microglia, stained using antibody against iba1, were also activated in the Mfn2 cKO mice at 12 weeks of age (Fig. 7c and Additional file 1: Figure S3), with Type IV amoeboid microglia appearing in the peripheral CA1 areas just adjacent to the subiculum, then encompassing the remaining CA1 by 18 weeks of age, (Fig. 7c) and in the cortical areas (Fig. 7e), which correlates with loss of neurons detected with antibody NeuN (Fig. 5a). By 28 weeks of age, activated microglia are absent in the CA1 (Fig. 7c, e), likely since most neurons are gone, yet GFAP-positive astrocytes continue to accumulate (Fig. 7b, d). No apparent microglia activation is observed in the CA2 regions at all ages examined.

### Mfn2 knockout caused cytoskeletal alterations

**Fig. 8:**
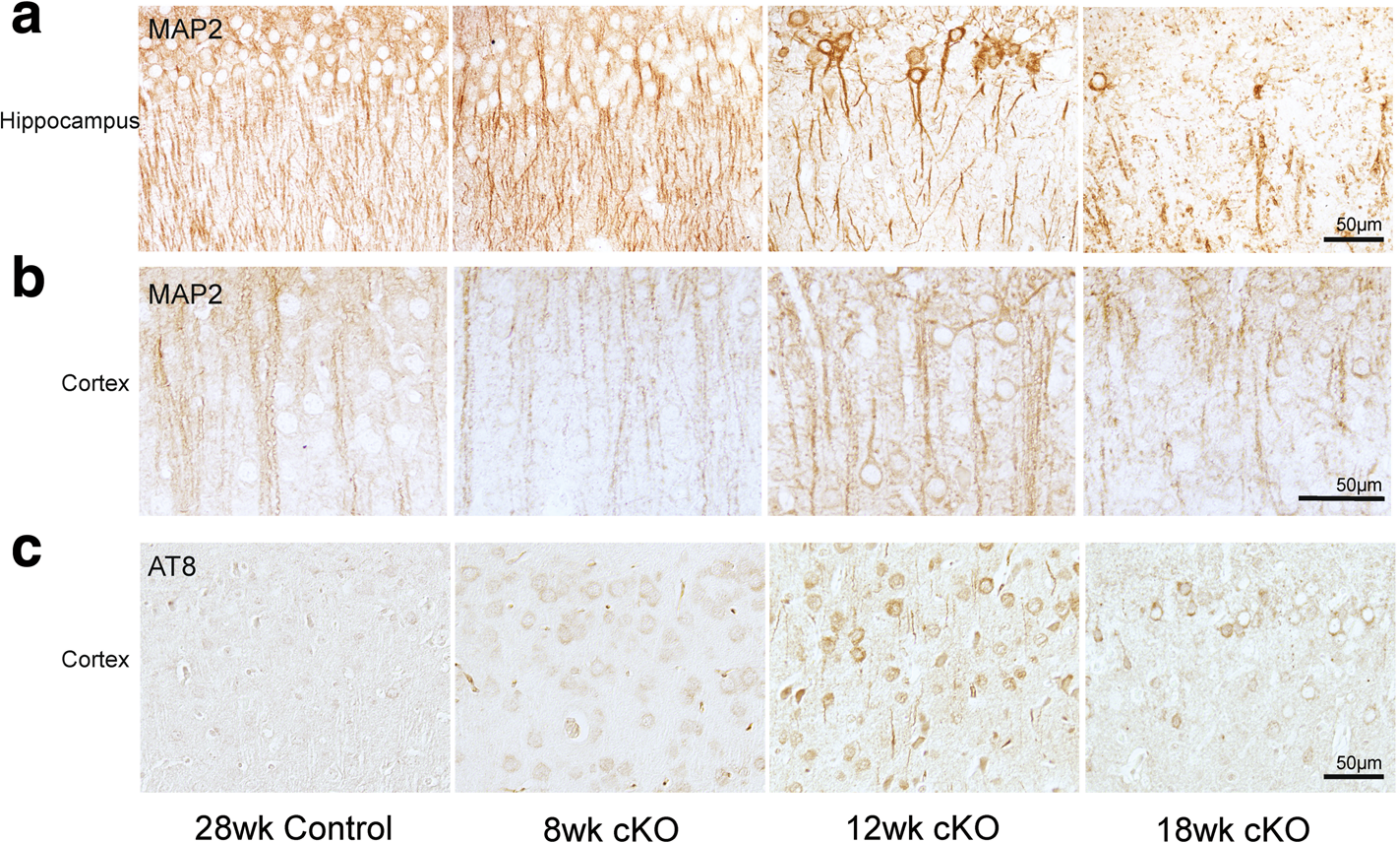
Mfn2 ablation caused abnormal cytoskeletal alterations in hippocampus and cortex in vivo. Representative immunohistochemistry of anti-MAP2 antibody in the hippocampus (**a**) and cortex (**b**) of Mfn2 cKO and control mice. Representative immunohistochemistry of anti-AT8, an antibody specifically against phosphorylated tau, in the cortex (**c**) of Mfn2 cKO and control mice

Cytoskeletal changes were also apparent in the neurons where Mfn2 was ablated with age (Fig. 8). In the hippocampus, MAP2 is present in CA1 neuronal dendrites, with a diffuse stain dispersed evenly in the cell bodies in control mice through all ages examined. Similar staining pattern of MAP2 was noted in Mfn2 cKO mice only up to the age of 8 weeks (Fig. 8a). By 12 weeks, however, the cellular distribution of MAP2 is changed dramatically in the CA1 region, such that there is a significant loss of dendritic staining, which correlates with increased localization of MAP2 in the cell body in Mfn2 cKO mice. This pattern remains until age 18 weeks, when very little MAP2 immunoreactivity remains in either the process or cell bodies (Fig. 8a and Additional file 1: Figure S3). Throughout the cortex, with increasing age, neurons in the Mfn2 cKO animals showed increasingly stronger cell body staining, with concomitant thickening of the processes (Fig. 8b). Higher levels of hyperphosphorylated tau, stained using the AT8 antibody, are found in the cortical neurons in 8 week Mfn2 cKO mice compared to the control mice. The AT8 immunostaining becomes more prominent in 12 week cKO mice with increased staining in the neuronal processes (Fig. 8c). In the CA1 region, only a few AT8-positive neuronal processes are found in the 8 week cKO, with weak staining in cell bodies at 12 weeks, and no immunoreaction by 18 weeks, correlating with the almost complete hippocampal neuronal loss (not shown).

## Discussion

Studies from multiple groups suggest that mitochondrial fragmentation largely due to disrupted mitochondrial fusion contributes to mitochondrial dysfunction and neuronal deficits in AD. To gain a better understanding of whether and how mitochondrial fragmentation causes AD-related deficits in the hippocampal and cortical neurons, in this study, we developed a transgenic mouse model of disturbed mitochondrial fusion in the pyramidal neurons of hippocampus and cortex by tissue-specific knockout of Mfn2. Interestingly, we found that ablation of Mfn2 caused a series of pathological events including mitochondrial damage and dysfunction, oxidative stress, inflammatory response, and cytoskeletal changes that eventually led to significant neurodegeneration in the hippocampus and cortex in vivo.

In our study, mitochondrial fragmentation became apparent in the CA1 hippocampal neurons since 8 weeks of age as reflected by significantly decreased mitochondrial aspect ratio. This was accompanied by increased ultrastructural damage to mitochondria as reflected by the loss of integrity of the internal structures. In older cKO mice (i.e., 28 weeks of age), swollen mitochondria with significantly enlarged size (i.e., doubled area) and almost complete round shape (aspect ratio close to 1) demonstrated almost complete loss of cristae, suggesting severe damage to mitochondria, a phenotype that was also observed in older DLP1 KO mice [28] and in the Mfn2 KO Purkinje Cells [48]. Mitochondrial function is dependent on their intact structure. Indeed, increased oxidative stress and impaired mitochondrial function such as reduced expression of OXPHO proteins and decreased mitochondrial respiratory parameters were also first observed in the Mfn2 cKO mice at 8 week of age when ultrastructural damage was observed. While the causal relationship between mitochondrial dysfunction and damage, and oxidative stress remains to be teased out, it was suggested that excessive mitochondrial fission leads to reduced mtDNA copy number [29] which could lead to reduced expression of essential OXPHOS proteins as we confirmed by the western blot analysis and cause mitochondrial dysfunction. Prior studies from multiple groups demonstrated that excessive fission or fusion caused deficits in complex assembly [30–32] likely through changes in the cristae shape and curvature or other uncharacterized mechanism that also negatively impacted mitochondrial function. Indeed, dramatic changes in the cristae organization was noted in the 8 weeks old animals. Mitochondrial dysfunction unavoidably causes increased oxidative stress which could damage mitochondrial ultrastructure and further exacerbate mitochondrial dysfunction. The progression of mitochondrial ultrastructural damage from relatively mild changes such as multilamellar appearance and vacuolation with partially broken cristae in Mfn2 cKO at 8 weeks of age to more severe appearance such as the extreme loss of internal cristae structure and significantly swollen mitochondria likely reflected the accumulation of oxidatively damaged mitochondria along the course. It is of importance to note that no mitochondrial defects were noted in the brain of the Mfn2 cKO mice at 4 weeks of age which excludes the potential complication due to developmental abnormalities. These results clearly demonstrated that Mfn2 ablation-induced mitochondrial fragmentation caused mitochondrial structural damage and dysfunction in the hippocampus and cortex in vivo.

The most striking change in the Mfn2 cKO mice is the apparent neurodegeneration in the hippocampal area at 18 weeks of age where near 90% of the CA1 hippocampal neurons were wiped out. While this extreme loss of neurons was not apparent in individual fields of the cortical area, taken together with the progressive shrinkage of the entire cortex with age, significant cortical neuron loss was apparent at 18 weeks of age when around 25% neurons were lost. Indeed, TUNEL assay did reveal that these neurons die by apoptosis in both the hippocampus and cortex at 18 weeks of age. Significant cortical neuronal loss continues to progress and reached around 50% at 28 weeks of age. It was noted that disabling mitochondrial function could produce the same pathological changes. For example, malonate treatment, which inhibits succinic dehydrogenase, resulted in strikingly similar hippocampal neurodegeneration [33]. Given that mitochondrial dysfunction far precedes neurodegeneration in this Mfn2 cKO model, our results clearly demonstrated that disrupted mitochondrial fusion could lead to neurodegeneration in AD-affected brain areas through mitochondrial dysfunction.

Interestingly, Mfn2 ablation induced neurodegeneration in the hippocampus and cortex is preceded by a series of pathological events in temporal order that may suggest a causal relationship between these events. Increased oxidative stress was observed in the Mfn2 cKO mice at the age of 8 weeks. It must be noted that oxidative stress is also a prominent and early feature of AD [34]. For example, advanced glycation end-products, lipid peroxidation, protein nitration and carbonyl formation have all been found to be increased in the brain of AD [34]. Further induction of HO-1 was determined to be a relatively early neuronal response as its appearance co-localized with the Alz50 tau epitope in degenerating neurons in AD [35]. At 12 weeks of age, cytoskeletal and neurofibrillary changes became apparent in the Mfn2 cKO mice. As temporally later events, cytoskeletal and neurofibrillary changes likely lie downstream of mitochondrial dysfunction and/or increased oxidative stress. In this regard, ample evidence demonstrated that oxidative stress could impact the posttranslational modification and lead to increased proteolysis of microtubule associated proteins, reduce the ability of microtubule to polymerize and cause severing of actin microfilaments and thus impair cytoskeletal structure in both neuronal and non-neuronal cells [36–38]. Soluble Aβ oligomers has been shown to cause proteolysis of microtubule associated proteins including (MAP2) during apoptosis [39]. Similarly, oxidative stress causes increased tau phosphorylation and promotes the conformational changes and fibrillation/aggregation of tau protein which leads to AD-related neurofibrillary changes [35, 40–42]. An inflammatory response was also observed in the Mfn2 cKO mice at 12 weeks of age which likely is also caused by mitochondrial dysfunction and/or oxidative stress. The inflammatory response, as shown by increased activation of microglia and astrocytes, precedes the observed neurodegeneration at 18 weeks of age. There are many studies that suggest inflammation as an early sign of AD disease progression. An inappropriate immune response may promote AD by increasing the production of Aβ and reducing removal of amyloid plaques by microglia [43]. Overall, our study demonstrated that mitochondrial fragmentation caused by disruption in mitochondrial fusion could initiate a cascade of abnormal changes that are relevant to the important pathological changes during the course of AD and lead to neurodegeneration in the hippocampus and cortex in vivo.

In addition to mitochondrial fragmentation and ultrastructural damage, we found an abnormal distribution of mitochondria, especially in the neuronal process, in the pyramidal neurons of Mfn2 cKO mice months before neurodegeneration. Loss of Mfn2 leads to decreased number of mitochondria in the axon and dendritic processes. In this regard, it is important to note that such an abnormal distribution of mitochondria was also noted in the brain of AD patients [9] and AD animal models [19]. This observation replicated prior findings where Mfn2 deletion also caused a prominent defect in mitochondrial content and transport in the processes of dopaminergic neurons which set DA neurons to degenerate 1 to 2 months later in retrograde manner [44, 45]. While it is not clear whether such abnormal mitochondrial distribution contribute to neurodegeneration in the hippocampal and cortical neurons in Mfn2 cKO mice since some neuron population could survive almost complete loss of mitochondria from its processes [46], it could contribute to neuronal dysfunction such as synaptic deficits of importance to AD as we demonstrated before [9]. Although altered expression of various mitochondrial fission/fusion proteins could differentially impact mitochondrial transport [12], Mfn2 may impact mitochondrial transport through its direct interaction with Miro and Milton [47], adaptor proteins crucial for kinesin-mediated transport of mitochondria.

As we demonstrated in this study, CA1 neurons are susceptible to Mfn2 loss. However, these neurons are resistant to DLP1 loss [25]. It is of interest to note that, in the contrary, dopaminergic neurons and Purkinje cells are susceptible to both the loss of Mfn2 and DLP1 [45, 46, 48–50], respectively. It is believed that different cells, including various neuron populations, have very different mitochondrial morphology according to their specific metabolic needs, and hence possess unique balance on the regulation of mitochondrial dynamics. Therefore, such a difference in the tolerance of loss of Mfn2 and DLP1 suggests that, comparing to other neuron populations, CA1 neurons more critically depend on mitochondrial fusion than fission for their function and survival which makes mitochondrial fusion a better target for restoring mitochondrial dynamic balance in AD. Alternatively, Mfn2 has been implicated in the regulation of mitochondrial properties besides fusion such as mitochondrial transport [47]. This could point to a difference in mitochondrial distribution to explain the preferential requirement of Mfn2 in the hippocampus for neuronal survival. However, this appears unlikely since studies of DLP1 loss in dopaminergic neurons show a similar abnormality in mitochondrial movement suggesting that DLP1 may also play a role in mitochondrial transport [46]. In this regard, the loss of DLP1 in the hippocampus increased the distance between mitochondria in the dendritic processes which was well tolerated, although overall content and number were unchanged [25].

## Conclusions

Overall, in this study we demonstrated that Mfn2-ablation induced mitochondrial fragmentation led to neurodegeneration through mitochondrial dysfunction and increased oxidative stress and a series of event in a strict temporal order in mice and all these pathological changes are also characteristics seen in AD during the course of disease. These results do not necessarily suggest that the pathological events occur at a similar temporal order in the AD brain, however, they suggest that disrupted mitochondrial dynamics and mitochondrial dysfunction could contribute to these pathological events in AD.

## Additional file

Additional file 1: Figure S1. Representative western blot and quantification analysis of brain homogenates found the DLP1 and OPA1 protein levels were unchanged in Mfn2 cKOmice (8 weeks of age) compared to control mice in both the hippocampus and the cortex. GAPDH was used as an internal loading control (*N* = 3/group, data represent mean ± SEM, Student’s t-test, **P* < 0.05). **Figure S2.** Nissl staining of the entire cortex showing neuronal layers I-VI (upper panels) and higher magnification of area encompassing layers III/IV (lower panels). Dashed line represents lower boundary of cortical neurons. Cortical shrinkage is apparent, neuronal layers become less distinct, and nuclei become disfigured and less uniform with age in the cKOmice. **Figure S3.** Quantification of the immunostaining of (A) GFAP, (B) IBA-1 and (C) MAP2 in the CA1 regions of the Mfn2 cKO mice at different ages (8–28 weeks) compared to control mice at 28 weeks of age (Data represent mean ± SEM, Student’s t-test, **P* < 0.05). (PDF 427 kb)
