# Supplementary material for: Mfn2 ablation causes an oxidative stress response and eventual neuronal death in the hippocampus and cortex
Source: Mol Neurodegener. 2018 Feb 1;13:5. doi: 10.1186/s13024-018-0238-8 (PMC5796581; doi:10.1186/s13024-018-0238-8)
Supplement: Additional file 1: Figure S1. — Representative western blot and quantification analysis of brain homogenates found the DLP1 and OPA1 protein levels were unchanged in Mfn2 cKOmice (8 weeks of age) compared to control mice in both the hippocampus and the cortex. GAPDH was used as an internal loading control (N = 3/group, data represent mean ± SEM, Student’s t-test, *P < 0.05). Figure S2. Nissl staining of the entire cortex showing neuronal layers I-VI (upper panels) and higher magnification of area encompassing layers III/IV (lower panels). Dashed line represents lower boundary of cortical neurons. Cortical shrinkage is apparent, neuronal layers become less distinct, and nuclei become disfigured and less uniform with age in the cKOmice. Figure S3. Quantification of the immunostaining of (A) GFAP, (B) IBA-1 and (C) MAP2 in the CA1 regions of the Mfn2 cKO mice at different ages (8–28 weeks) compared to control mice at 28 weeks of age (Data represent mean ± SEM, Student’s t-test, *P < 0.05). (PDF 427 kb) [file 13024_2018_238_MOESM1_ESM.pdf]

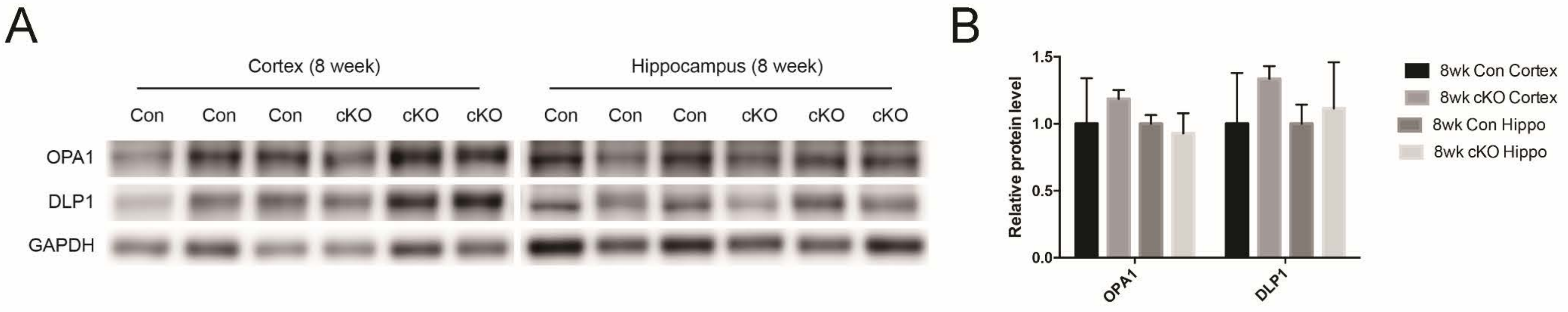

Figure S1. Representative western blot and quantification analysis of brain homogenates found the DLP1 and OPA1 protein levels were unchanged in Mfn2 cKO mice (8 weeks of age) compared to control mice in both the hippocampus and the cortex. GAPDH was used as an internal loading control (N=3/group, data represent mean ± SEM, Student's t-test, *P < 0.05).

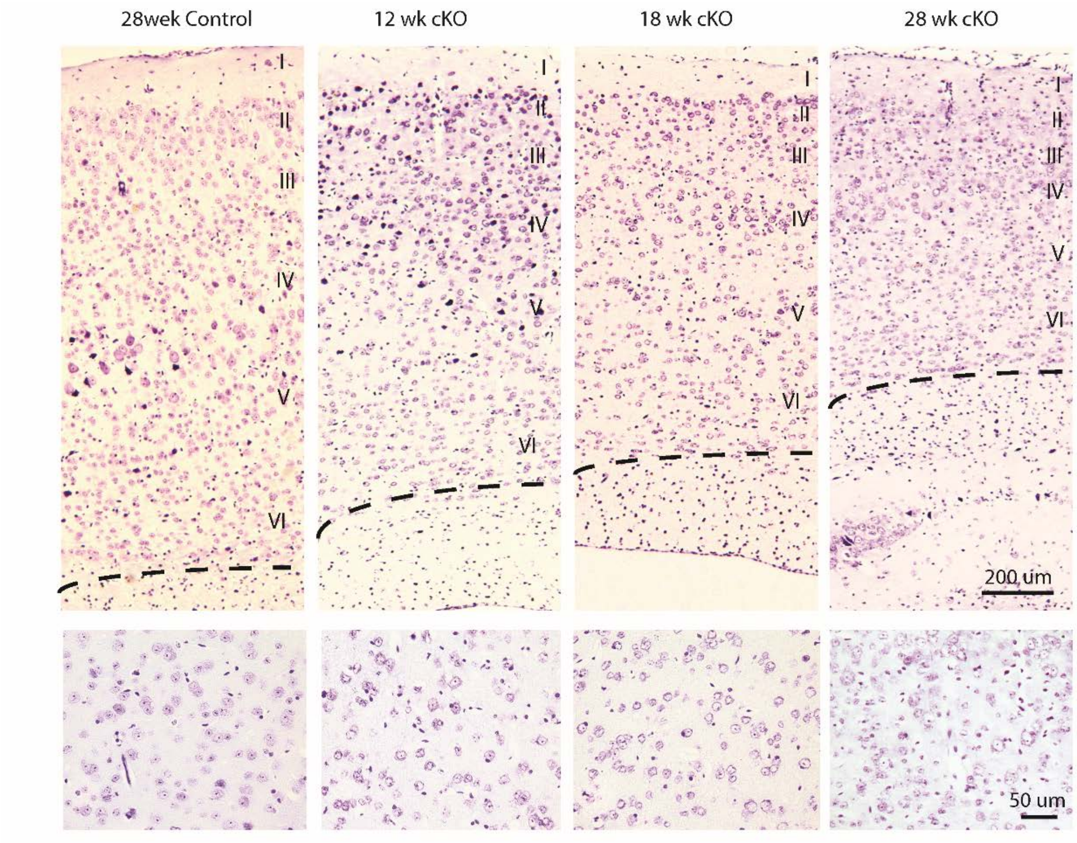

Figure S2. Nissl staining of the entire cortex showing neuronal layers I-VI (upper panels) and higher magnification of area encompassing layers III/IV (lower panels). Dashed line represents lower boundary of cortical neurons. Cortical shrinkage is apparent, neuronal layers become less distinct, and nuclei become disfigured and less uniform with age in the cKO mice.

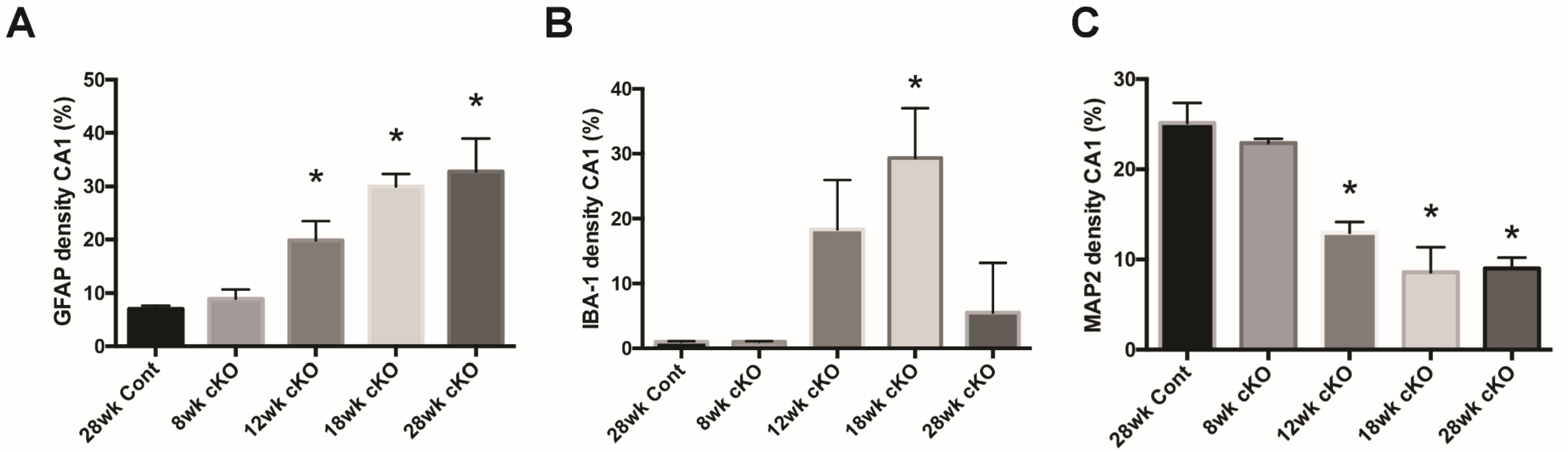

Figure S3. Quantification of the immunostaining of (A) GFAP, (B) IBA-1 and (C) MAP2 in the CA1 regions of the Mfn2 cKO mice at different ages (8-28 weeks) compared to control mice at 28 weeks of age (Data represent mean ± SEM, Student's t-test, *P < 0.05).
